# The extracellular matrix in the kidney: a source of novel non-invasive biomarkers of kidney fibrosis?

**DOI:** 10.1186/1755-1536-7-4

**Published:** 2014-03-28

**Authors:** Federica Genovese, Alba A Manresa, Diana Julie Leeming, Morten Asser Karsdal, Peter Boor

**Affiliations:** 1 Nordic Bioscience, 2730 Herlev, Denmark; 2 Nephrology and Immunology, RWTH Aachen University, Aachen, North Rhine-Westphalia, Germany; 3 Institute of Pathology, RWTH Aachen University, Aachen, North Rhine-Westphalia, Germany; 4 Institute of Molecular Biomedicine, Comenius University, Bratislava, Slovakia

**Keywords:** Kidney fibrosis, Biomarkers, Extracellular matrix, Matrix metalloproteinases

## Abstract

Interstitial fibrosis is the common endpoint of end-stage chronic kidney disease (CKD) leading to kidney failure. The clinical course of many renal diseases, and thereby of CKD, is highly variable. One of the major challenges in deciding which treatment approach is best suited for a patient but also in the development of new treatments is the lack of markers able to identify and stratify patients with stable versus progressive disease. At the moment renal biopsy is the only means of diagnosing renal interstitial fibrosis. Novel biomarkers should improve diagnosis of a disease, estimate its prognosis and assess the response to treatment, all in a non-invasive manner. Existing markers of CKD do not fully and specifically address these requirements and in particular do not specifically reflect renal fibrosis. The aim of this review is to give an insight of the involvement of the extracellular matrix (ECM) proteins in kidney diseases and as a source of potential novel biomarkers of renal fibrosis. In particular the use of the protein fingerprint technology, that identifies neo-epitopes of ECM proteins generated by proteolytic cleavage by proteases or other post-translational modifications, might identify such novel biomarkers of renal fibrosis.

## Review

Renal fibrosis is the principal pathological process underlying the progression of chronic kidney disease (CKD) and finally leading to end-stage renal disease (ESRD). For patients progressing to ESRD the mortality levels exceed those of some malignancies. This devastating condition is not only a major problem for the lives of patients, but also an economic burden for the health system.. The *US Renal Data System, USRDS 2013 Annual Data Report* estimated that 14% of the adult population in the USA had CKD and the costs for CKD patients older than 65 reached over $ 45 billion [1]. Patients with ESRD require lifelong dialysis and the only possible treatment is kidney transplant.

Renal and in particular interstitial fibrosis is a common feature of CKD, regardless of the etiology of the primary disease. Interstitial fibrosis is the strongest indicator of disease progression, even when the primary disease is of glomerular origin [2]. Therapies for renal fibrosis with proven efficacy in clinical settings currently do not exist. The challenge in finding anti-fibrotic therapies is partly due to the need of long and expensive clinical trials, as the currently used clinical endpoints require long study durations and a large number of patients [3]. The development of novel, non-invasive, fibrosis-specific biomarkers, reflecting morphological tissue changes at early stages and predicting the evolution of renal fibrosis, would be of great importance. Such biomarkers would facilitate clinical studies with experimentally established drugs targeting profibrotic molecules and could identify patients that need to be treated at the right moment.

The PubMed database was searched to identify articles on renal fibrosis using the following keywords: renal fibrosis, extracellular matrix (ECM), CKD, biomarkers, collagen, proteoglycans, glomerular basement membrane, mesangium and matrix metalloproteinase (MMP), as Medical Subject Headings (MeSH). The reference lists of identified papers were also used for further search. Each author further selected key publications based on their personal knowledge on the topic of biomarkers for renal fibrosis. Only full-text articles written in English were included and the focus was placed on studies published within the last three years.

### Mechanisms of renal fibrosis

Renal fibrosis, that is, the accumulation and dysregulated remodelling of ECM, can affect all major compartments of the kidney being termed glomerulosclerosis in the glomeruli, tubulointerstitial fibrosis in the tubulointerstitium and arterio- and arteriolosclerosis in the vasculature. At a certain point, virtually all renal cells are involved in fibrosis [4]. The description of the cellular and molecular mechanisms of kidney fibrosis is beyond the scope of this review and has already been thoroughly discussed by others [5-7]. We will focus on the mechanisms related to ECM accumulation and remodelling in renal fibrosis as a potentially relevant source of novel biomarkers for renal fibrosis.

**Figure 1:**
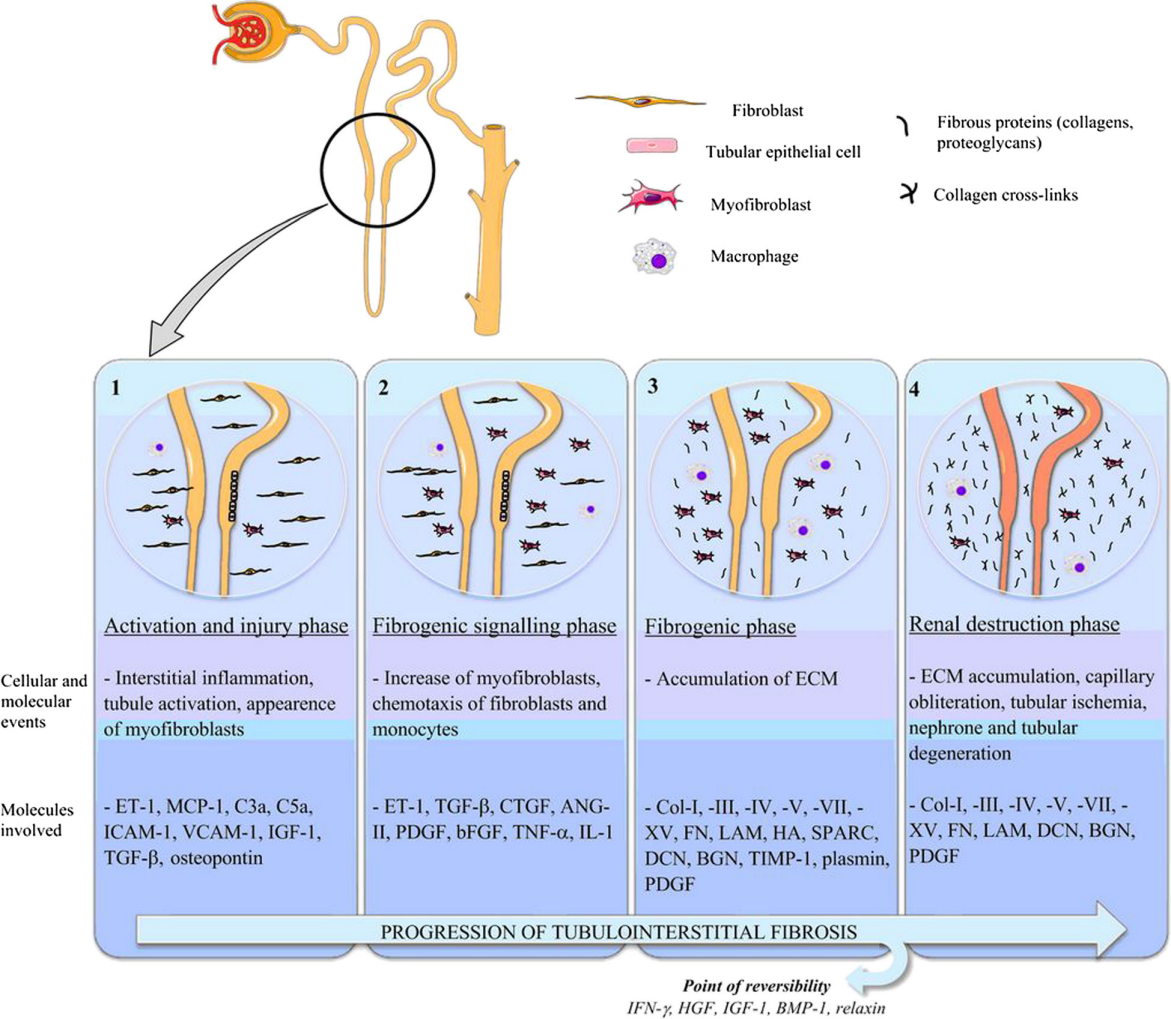
**Progression of renal interstitial fibrosis.** Fibrogenesis starts with an initial tissue injury that causes inflammation as the physiological host defense response. When this response becomes uncontrolled and sustains itself with continuous production of chemotactic cytokines, inflammation does not resolve and can create the optimal microenvironment for tissue fibrogenesis.

Renal fibrosis is the result of a failed wound healing process that occurs after an initial insult. The pathophysiology of renal fibrosis can be divided into four phases: 1) cellular activation and injury phase or priming; 2) fibrogenic signalling phase or activation; 3) fibrogenic phase or execution; and 4) destructive phase or progression. Figure 1 describes the different phases of tubular interstitial fibrosis and some of the cells and molecules that intervene in the process. These phases can be best studied and differentiated in animal models, in which a disease stimulus is often applied at a single time-point so that the injury and the progression are synchronized. In most, if not all, human diseases this is not the case and, to a variable and yet not defined extent, all phases can be observed at the same time. Various mediators of renal fibrosis have been described, such as the prototypical profibrotic molecules transforming growth factor beta 1 (TGF-β1) and platelet-derived growth factor (PDGF), which will not be discussed in detail here [8,9]. Among the effectors causing a pathological matrix accumulation, plasminogen activator inhibitor-1 (PAI-1), which is induced by TGF-β, was shown to modulate fibrosis via effects on cell migration, matrix turnover and macrophage infiltration [10]. The role of this effector in kidney fibrosis has been described elsewhere [11]. Even though many cell types in the kidney are able to produce ECM, (myo-)fibroblasts in the interstitium and mesangial cells in the glomeruli are considered the main cellular mediators of interstitial fibrosis and glomerulosclerosis, respectively [2,12]. In the kidney, myofibroblasts can originate from different sources, the most important being resident interstitial fibroblasts in the cortex and pericytes in the medulla. Other sources seem to contribute to a lesser and varying extent to the pool of myofibroblasts and include endothelial cells (via endothelial-to-mesenchymal transition), tubular epithelial cells (via epithelial-to-mesenchymal transition) and fibrocytes [2,12,13].

Fibronectin is the first ECM protein that is deposited in fibrogenesis [14]. It activates integrins, functions as a fibroblast chemoattractant and co-localizes with collagen formation. This triggers the production of a large variety of ECM proteins, discussed below [7]. The synthesis, deposition and degradation of different ECM proteins, their post-translational modifications, together with the induction of proteases and protease inhibitors and other ECM remodelling enzymes (for example tissue transglutaminase) contribute to the development of irreversible fibrosis [7].

### Diagnosis of renal fibrosis

At present, kidney biopsy is the only method to detect renal fibrosis. It is an invasive procedure with possible complications. The extent of interstitial fibrosis in kidney biopsy is most often reported in a semi-quantitative manner and has several intrinsic limitations, mainly due to sampling error and to intra- and inter-observer variability [15]. Imaging techniques, such as ultrasound, can show signs of corticomedullary differentiation, which is a sensitive but not specific marker of CKD; it can moreover show the size of the kidneys, the presence of cysts and solid lesions, urinary obstruction or scars but it cannot diagnose the presence of ongoing interstitial or glomerular fibrogenesis [16]. Another imaging technique that is attracting increasing interest is the magnetic resonance elastography (MRE), already used in the hepatic field to detect liver fibrosis [17]. MRE can non-invasively sample tissue stiffness *in vivo*, and its possible use in renal fibrosis is under evaluation [18]. At the moment, there are no specific molecular imaging modalities for renal fibrosis.

Serological and urinary markers can rapidly change following a physiological or pathological event, and are therefore dynamic. Here we will discuss established, developing and potential serological and urinary markers of renal fibrosis.

### Chronic kidney disease (CKD) biomarkers

In the last decade, there has been intense interest and effort in finding novel predictive biomarkers for the diagnosis and prognosis of CKD. Several molecules involved in kidney function, signalling and structure have been evaluated as potential markers for CKD [19]. The only markers currently accepted and used in clinical practice for the diagnosis and prognosis of CKD are markers of loss of kidney function. The most widely used are the estimated glomerular filtration rate (eGFR) [20], serum creatinine, blood urea nitrogen (BUN) [21] and albuminuria or proteinuria [22]. Cystatin C [23] and β-trace protein [24] have been proposed as an alternative to creatinine to estimate the GFR. These markers indicate impaired renal function but have no disease specificity, and detectable changes in their concentration come after the biological changes in the organ causing the functional impairment.

Molecules involved in inflammation or in signalling leading to the onset of fibrosis have been studied as possible markers for renal fibrosis. Some of these molecules belong to the panel of urinary biomarkers proposed by the Predictive Safety Testing Consortium (PSTC) for the detection of drug-induced kidney toxicity [25]. Even though the purpose of these markers is to detect an acute response to the injury, some are also being evaluated as early markers of CKD and progression towards ESRD. These molecules include: C-reactive protein (CRP), tumor necrosis factor receptor II (TNFRII), TGF-β1 and pentraxin-3 as cytokines involved in the development of CKD; asymmetric dimethylarginine (ADMA) as a marker of endothelial dysfunction and consequent kidney damage [26]; fibroblast growth factor-23 (FGF-23), adiponectin and apolipoprotein A-IV as metabolic factors involved in the regulation of kidney metabolism; and gamma-glutamyl transpeptidase (GGT) as molecules involved in oxidative stress, which can contribute to CKD pathogenesis. Endostatin, the N-terminal portion of collagen type XVIII, is a potent anti-angiogenic factor which has been recently evaluated as a marker of CKD. A significant elevation of endostatin in plasma of patients with CKD following disease severity compared to controls without CKD was observed [27]. Except FGF-23, all the other markers are not kidney-specific and require further evaluation in larger clinical cohorts to confirm their potential (reviewed thoroughly elsewhere [19]). FGF-23 is currently one of the most promising markers for CKD. This phosphaturic hormone is increased in serum in a physiological adaptation to the hyperphosphatemia that arises when the GFR decreases below 25 ml/min/1.73 m^2^[28]. Several studies demonstrated the potential of FGF-23 as a marker of mortality in dialysis patients [29], initiation of chronic dialysis [30], CKD progression [31,32], cardiovascular disease [30], cardiovascular mortality [32] or all-cause mortality [30,32].

Kidney-specific molecules are more likely to specifically reflect renal injury. Such molecules include podocyte-specific proteins nephrin, podocin and podocalyxin as urinary markers of glomerular damage [33,34]. Following the same rationale, neutrophil gelatinase-associated lipocalin (NGAL) [35], kidney injury molecule-1 (KIM-1) [36,37], N-acetyl-beta-D-glucosaminidase (NAG) and liver-type fatty acid-binding protein (L-FABP) [38-41] can be markers of tubular damage, as these are proteins expressed in the tubules that can be released in serum or urine following tubular damage. Both NGAL and KIM-1 are well-known markers of acute kidney injury and their potential as diagnostic and prognostic markers of CKD has been evaluated in various studies and reviewed in detail elsewhere [19]. NGAL was shown to be increased in serum and/or urine of patients suffering from different kidney diseases, for example in patients with IgA nephropathy (IgAN), various glomerulonephritis, autosomal dominant polycystic kidney disease (ADPKD), pediatric lupus nephritis and CKD from a range of etiologies, and to differentiate between CKD stages [19,42]. NGAL might be a good marker for tubulointerstitial injury in CKD, and might identify progression of the disease. It has to be mentioned though, that the results are not consistent in all studies [35,43,44]. Urinary KIM-1 levels were associated with the outcome of incident CKD or rapidly declining kidney function in the Multi-Ethnic Study of Atherosclerosis (MESA) cohort [44]. Other studies [41,43,44] showed a good potential for KIM-1 as a diagnostic marker for CKD and even as a marker of efficacy of intervention. However, as for many other markers, confirmation in long-term observational studies using larger populations is still required [19,43]. First hints suggest that cytokeratin 18, which can be released into urine and circulation following renal epithelial cell death, might also be a novel marker of CKD. Serological and urinary concentration levels of total cytokeratin 18 measured in CKD patients could separate patients with advanced CKD from patients with mild disease and healthy controls [45]. All these molecules have been evaluated for their association with impaired kidney function, but they are not directly linked to fibrosis, that is, to the deposition and remodelling of ECM. The tubular damage markers are not completely specific for the kidneys, as many of these proteins are also involved in other diseases, as for example NGAL (also known as lipocalin-2) in the liver.

In the search for specific biomarkers of kidney fibrosis, the ECM proteome is a large source of new potential targets. Only a few of these proteins have been analyzed as diagnostic and prognostic markers, despite their involvement in renal fibrosis that has been proven. A good biomarker should reflect the presence of renal fibrosis and be linked to an outcome (decline in eGFR, renal failure, death). The ideal biomarker should be detected non-invasively and should be able to predict the progression of the disease and/or the response to a treatment in a more sensitive and specific manner compared to standard parameters.

The following sections give an overview on involvement of renal ECM proteins and proteases in renal fibrosis and their potential utility as diagnostic or prognostic markers of renal fibrosis.

### The extracellular matrix (ECM) of the kidney

The ECM is a very dynamic, highly charged structure which acts both as a support structure for the cells and as an active component in cell signalling [46]. It is composed of collagens, glycoproteins and elastin molecules which form a complex network interacting with each other and with the surrounding cells. Proteases, for example MMPs and their inhibitors, are responsible for maintaining the equilibrium between formation and degradation of ECM proteins. In the kidney cortex, the ECM is present in anatomically distinct areas with different functions depending on its molecular components:

1. in the glomeruli

a. glomerular basement membrane

b. Bowman’s capsule

c. mesangial ECM

2. in the tubulointerstitium

a. tubular basement membrane (in part segment-specific)

b. peritubular capillary basement membrane

c. interstitial ECM

3. in larger vessels

a. within the vessels (lamina elastica interna and externa)

b. around the vessels (adventitia of arteries and veins)

Medullary interstitial ECM is physiologically more prominent compared to the cortical interstitial ECM, steadily increasing in quantity in the direction from outer to inner medulla/papilla. The functional consequence of this difference is yet unclear. The hilar region, renal pelvis (for example suburothelial basement membrane) and renal capsule are also composed of ECM. The contribution and remodelling of ECM in these specific anatomical locations in renal fibrosis are not well-studied.

The next paragraphs describe the proteins that compose the renal ECM in healthy state and those involved in the onset of fibrosis, with particular focus on those that can be a source of new biomarkers. A comprehensive list of experimental evidence for ECM proteins being involved in renal fibrosis, derived from both pre-clinical and clinical studies, is included in Additional file 1: Table S1.

### Glomerular basement membrane (GBM)

The glomerular basement membrane (GBM) is thicker compared to most other basement membranes. It contains four main macromolecules: laminin, collagen type IV, nidogen and heparan sulphate proteoglycans. The main function of the GBM is to act as a charge- and size-selective filtration barrier between the vascular system and the urinary space.

Laminin is secreted as an αβγ heterotrimer (α5, β2 and γ1 laminins are present in the mature GBM [47]), which forms a network required to maintain the basement membrane integrity. Mutations of the laminin genes can lead to kidney diseases, for example mice with a hypomorphic mutation in the gene for the laminin α5 subunit develop polycystic kidney disease [48]; a null mutation of the gene for the laminin α4 subunit can cause progressive glomerular and tubulointerstitial fibrosis [49]; and truncation or severe missense mutations in the gene for the laminin β2 subunit can cause Pierson syndrome, characterized by premature death from renal failure [47].

Collagen type IV is composed by three α chains that fold in a triple helix and, by binding with other collagen type IV molecules, form the meshwork conformation typical of the basement membrane. The α3(IV), α4(IV) and α5(IV) chains are the most expressed in the adult GBM [50]. Mutations in the gene for the α5 chain of collagen type IV cause the X-linked Alport syndrome in humans, a rare genetic disease characterized by progressive glomerular injury. Mutations in the genes for the α3 and α4 chains can cause autosomal recessive and autosomal dominant Alport syndrome and thin basement membrane nephropathy. Collagen type IV is also the target of two autoimmune diseases affecting the kidney: Goodpasture’s syndrome and Alport post-transplantation disease. Both diseases are characterized by autoantibodies attacking the GBM and causing rapidly progressive glomerulonephritis [47]. Knock-out mice for the gene for the α3 chain and for the α5 chain of collagen type IV are widely used as murine models of autosomal and X-linked Alport syndrome, respectively [51,52]. Increased collagen type IV expression was described in chronic transplant nephropathy using immunohistochemistry [53]. The distribution of up-regulated collagen type IV was uniform in the GBM, in the mesangium and in the interstitium. Collagen type IV was also used in experimental animal studies as a marker of glomerular sclerosis and interstitial fibrosis [8,54].

Elevated urinary concentration levels of collagen type IV have been associated with the decline of renal function in patients with type 1 [55] and type 2 diabetes [56,57], but also in non-diabetic nephropathies, such as membranous nephropathy and anti-neutrophil cytoplasmic antibody (ANCA)-associated glomerulonephritis [58]. Specifically, type 1 diabetic nephropathy (DN) patients with elevated urinary collagen type IV to creatinine ratio (T4C) but normal albumin to creatinine ratio (ACR) declined more rapidly in eGFR than patients with normal T4C [55]. In type 2 diabetic patients, increased collagen type IV urine excretion was associated with the severity of morphological alterations in fibrosis, albeit no direct relationship with the content of collagen type IV in the kidney could be observed [56]. Another study in type 2 diabetic patients with normoalbuminuria and microalbuminuria found an inverse correlation between urinary collagen type IV excretion and the outcome annual decline of eGFR, but no correlation with progression to advanced diabetic nephropathy was found [57]. In a study on biopsy-proven membranous nephropathy and ANCA-associated glomerulonephritis [58] elevated levels of urinary collagen type IV were correlated with urinary proteins, urinary NAG and selectivity index. Different results were observed in a urinary peptidome study performed in type I diabetic patients. Patients with progressive early function decline showed a decreased expression of fragments of collagen type IV (α1 chain) compared with control subjects with stable renal function [59]. These results suggest that urinary collagen type IV might be a promising additive biomarker in patients with diabetic nephropathy and further clinical studies are eagerly awaited.

The transmembrane collagen type XVII has been recently identified in the GBM. Its deficiency causes effacement of podocyte foot processes, therefore it might be involved in the attachment of the podocyte to the GBM [60]. Nidogen 1 and 2 bind to collagen type IV and laminin separately. Although nidogens have a role in the basement membrane formation, experimental evidence showed that they are not strictly required for GBM formation [61]. Agrin is the major heparan sulphate proteoglycan of the GBM in healthy kidneys, while perlecan is an abundant component of other basement membranes [62]. Perlecan expression levels are increased in the glomeruli of IgAN patients and correlate with a lower urinary albumin excretion, suggesting that perlecan could be a marker of slower progression of the disease, and therefore of better outcome [63]. Perlecan and agrin, as all the heparan sulphate proteoglycans, have highly negatively charged glycosaminoglycans (GAGs), assumed to contribute to the negative charge of the basement membrane [61]. Interestingly, several studies showed that lack of perlecan and agrin does not lead to proteinuria, even though it affects the negative charge of the GBM [64-66].

### Mesangial ECM

The mesangial ECM provides structural support for the glomerular capillary convolute, connecting with the extraglomerular mesangium at the vascular pole. It has a role in cell-matrix signalling in a bidirectional manner. Dysregulation of this cell-matrix signalling plays a role in a wide range of glomerular diseases [67], such as IgAN [68] and DN [69]. Mesangial ECM differs substantially from GBM, and its composition allows larger molecules to pass to the mesangium. In physiological conditions its major components are fibronectin, collagen type IV (α1 and α2 chains, but not α3 and α5), collagen type V, laminin A, B1 and B2, chondroitin sulphate and heparan sulphate proteoglycans (perlecan, collagen type XVIII and bamacan, but not agrin) and nidogen [47,67]. The small proteoglycans decorin, biglycan, fibromodulin and lumican are weakly expressed in the mesangial matrix and rather localized in the tubular interstitium [70]. Under pathological conditions, decorin and biglycan were shown to be up-regulated in glomeruli [63]. Consistently, elevation of collagen type IV was also reported in several studies with humans and rodent models where protein localization and both protein and mRNA levels were assessed [69,71-73].

Typical scar collagen type I is *de novo* expressed in glomerulosclerosis, and by inhibiting its accumulation, a reduction in the extent of glomerulosclerosis was obtained in a model of DN [74]. MMPs play an important role in the homeostasis of the mesangial matrix, for example alterations on MMP function were shown to be linked to light chain diseases [75].

At the moment it is unclear whether glomerular ECM (either GBM or mesangium) might provide specific biomarkers of glomerular injury.

### Interstitial ECM

The renal interstitial matrix is normally composed by collagen type I, III, V, VI, VII and XV, both sulphated and non-sulphated glycosaminoglycans, glycoproteins and polysaccharides. During fibrosis, the formation of scar tissue in the interstitial space is the result of the excessive accumulation of ECM components.

#### Collagens

Collagens constitute the main structural element of the interstitial ECM, providing tensile strength, regulating cell adhesion, support, chemotaxis, cell migration and tissue development [76]. Collagen type I and III are known to be deposited in early stages during renal fibrosis [73,77,78].

Collagen type I accumulates in fibrotic glomeruli, tubulointerstitial space and arterial walls in pathological conditions, co-localizing with decorin and biglycan [79]. Collagen type I accumulation in fibrosis, as many other ECM molecules, is both due to decreased degradation and elevated synthesis [14,69]. Urinary proteome analyses could differentiate DN patients from healthy individuals and patients with other chronic kidney diseases [80]. Among the proteins differentially expressed, fragments of collagen type I were significantly less present in the urine of DN patients. The authors suggested that this indicated a decreased collagen proteolysis, probably due to cross-linking rendering the collagens resistant to proteolytic cleavage or to increased protease inhibitor expression [80].

In physiological conditions collagen type III is normally expressed at low levels in the interstitium, and it is undetectable in glomeruli. However, during fibrosis the expression levels are increased in the interstitium and in the glomeruli, as shown by immunohistochemical analysis on human renal biopsies using antibodies against the collagen type III N-terminal pro-peptide (PIIINP) [81].

PIIINP was detected in high concentrations in urine and serum of patients with various renal diseases [81-83]. Urinary PIIINP (and collagen IV) levels were elevated in patients with various nephropathies and correlated with the extent of interstitial fibrosis in kidney biopsies [81]. The urinary PIIINP to creatinine ratio (uPIIINP/Cr) was evaluated in kidney transplant patients and correlated with the extent of interstitial fibrosis [82]. Furthermore, in another study on patients with different CKD stages subjected to kidney biopsy, uPIIINP/Cr correlated with serum creatinine, eGFR and CKD stage as well as with the extent of fibrosis evaluated in the biopsies [83].

Elevation of collagen type V and VI in kidney fibrotic tissue has been reported in various studies [69,84]. Conversely, decreased concentrations of collagen type V (α1 chain) were observed in a urinary peptidome of patients with type 1 DN with early renal function decline [59]. Although different types of collagen are highly up-regulated in renal fibrosis, so far only PIIINP and collagen type IV have been analyzed as potential biomarkers. Both are among the most promising specific markers reflecting renal fibrosis.

#### Glycoproteins

Fibronectin is an adhesive glycoprotein involved in the organization of the ECM. Its accumulation is one of the first events during renal fibrosis [14]. It was shown to be up-regulated in many animal models and in human CKD [69,71,73,78,85].

Thrombospondin-1 (TSP-1) is an adhesive glycoprotein involved in fibroblast proliferation and migration [86]. It was shown to be up-regulated before the disease onset, and correlated with the degree of tubulointerstitial fibrosis in three different rat models of renal fibrosis. TSP-1 was observed to be transiently expressed at early fibrosis stages, suggesting a possible role as a mediator of interstitial fibrosis via activation of TGF-β [69,86].

Proteoglycans are a subgroup of glycoproteins with a high content of carbohydrates, which fill the majority of the renal extracellular interstitial space. They have a wide variety of functions, such as hydration, force-resistance and growth factor binding [87]. The latter is important in renal fibrosis as proteoglycans act as a reservoir of profibrotic growth factors, such as the latent forms of TGF-β or FGF-2 [5].

Decorin, biglycan and fibromodulin are small leucine-rich proteoglycans (SLRPs), which act as potent regulators of TGF-β [62,88]. Decorin and biglycan also have an important role in collagen fibrillogenesis. In healthy adult renal tissue, decorin and biglycan are expressed in the tubulointerstitium and weakly in the glomeruli [69]. However, during progressive renal scarring an increased expression of decorin and biglycan was observed in various experimental models of renal injury and in humans [79,84,85,89-91]. Specifically, in the unilateral ureteral obstruction (UUO) model, tubular biglycan up-regulation was observed before macrophage infiltration, indicating that biglycan could act as an initiator and regulator of inflammation in the kidney [89]. Biglycan and decorin expression was also found to be highly up-regulated in the glomeruli of IgAN patients, indicating a potential role in this glomerular disease [63]. Decorin has well-known anti-fibrotic properties: it neutralizes TGF-β activity by interfering with its signalling; it exerts an anti-apoptotic activity on tubular epithelial and endothelial cells; and can induce fibrillin-1 expression, by binding the insulin-like growth factor type I (IGF-I) receptor [92]. Its use as an anti-fibrotic molecule has been shown in a rat model of glomerulonephritis [93].

Hyaluronan is a high molecular weight glycosaminoglycan formed by the repetition of disaccharides composed by N-acetylglucosamine and glucuronic acid [62]. It has the ability to bind to a variety of proteoglycans and to cell receptors acting as a signalling molecule. In a healthy human kidney, hyaluronan is very little expressed. However, during progressive kidney disease, it accumulates in the cortical interstitium and may potentiate interstitial inflammation by stimulating the recruitment of monocytes to the interstitial space [5]. Elevated levels of hyaluronan in renal tissue were reported in several kidney diseases in both rat models, for example ischemia-reperfusion injury, and human diseases, for example DN, renal transplant rejection and kidney stone formation [94].

Versican is a chondroitin sulphate proteoglycan, the largest member of the modular proteoglycans, with an important role in maintaining the integrity of the ECM by interacting with hyaluronan [89,95]. In healthy renal tissue, versican is found expressed in the tubulointerstitium and the blood vessels, but not in the glomeruli [96]. In patients with different proteinuric nephropathies, versican expression was increased in areas with marked tubulointerstitial fibrosis, suggesting that versican may have an important role during CKD progression [97]. Most of the above mentioned glycoproteins undergo significant regulation during kidney fibrosis. Still, no data exist on their potential as renal biomarkers.

### Matrix metalloproteinases (MMPs) and other proteases

The enzymes playing a central role in matrix remodelling are metalloproteinases. Metalloproteinases are synthesized in kidneys and have an important function in maintaining the homeostasis of the ECM. The main families of metalloproteinases are MMPs, a disintegrin and metalloproteinase (ADAM) proteins and a disintegrin and metalloproteinase with thrombospondin motifs (ADAMTS). The role of ADAMs and ADAMTS is only starting to emerge and will not be discussed here [98-103]. Serine proteases (plasmin and cathepsin G) and cysteine proteases (cathepsins B, H and L) can also contribute to the degradation of ECM components at neutral pH [104].

#### MMPs and tissue inhibitors of metalloproteinases (TIMPs)

MMPs are zinc-dependent enzymes involved in ECM remodelling, which play a central role in tissue homeostasis. There are 23 MMPs in humans [105] and at least ten of them are expressed in the kidney (MMP-1, -2, -3, -9, -13, -14, -24, -25, -27 and -28) [106]. MMP-12 was thought not to be expressed in the kidney even though some experimental results in an animal model suggest the opposite [107]. MMPs were hypothesized to be anti-fibrotic due to their function as ECM degradation enzymes. Increasing evidence suggests that MMPs have a more complex role in renal fibrosis [4,108,109]. For example, MMP-9-mediated degradation of collagens creates collagen fragments, which possess chemotactic properties for neutrophils and are able to stimulate MMP-9 production. Apart from their action on ECM components, MMPs are also known to modulate growth factors and their receptors (TGF-β, FGF-R1), adhesion molecules (integrins and cadherins) [109], cytokines and chemokines. Consequently, MMPs are involved in several processes aside from ECM remodelling, such as destruction of the basement membrane, angiogenesis, cell migration and cell apoptosis, some being pro- and some anti-fibrotic depending on the context [4,109,110]. MMPs are inhibited permanently by degradation or temporarily by tissue inhibitors of metalloproteinases (TIMPs). A balance between MMP and TIMP activity is essential for ECM homeostasis [109]. Among the four TIMPs that have been identified in vertebrates, TIMP-1, -2 and -3 are expressed in the kidney [106]. Increased mRNA and protein levels of TIMP-1 were reported in several human and rodent models of different renal diseases, suggesting that TIMP-1 might be involved in the early events during the progression of renal diseases [5,73,109]. TIMP-2 has also been shown to be elevated in various rat models of renal disease [73]. The exact localization and temporal expression of MMPs in the human kidney is still not completely understood [108]. Most of the data on MMP expression derive from animal models of kidney diseases (Additional file 1: Table S1). MMP-2 and MMP-9 are known to be involved in the proteolysis of collagen type IV, which accumulates in the basement membranes, for example in early stages of DN. MMP-2 and MMP-9 expression and activity were up-regulated in different animal models of renal fibrosis [69,91,109,111,112], but were decreased in cases of DN in both humans and rats [69,113]. Changes in MMP-2 and MMP-9 activity might therefore influence the ECM composition causing renal damage at early stages of DN [106]. However, another study showed that urinary levels of MMP-9, together with collagen type IV, were elevated in type 2 DN patients with macroalbuminuria [114]. MMP-3 expression and activity during DN was decreased in both humans and rats [69]. MMP-7 is not expressed in healthy human kidneys but was found in epithelial cells and atrophic tubules in patients with ADPKD and in a mouse model of acute renal tubule injury and chronic progressive renal fibrosis [115]. Some of the contrasting results, particularly in regards to MMP-2 and MMP-9, can be explained by the impossibility to distinguish between the active and the inactive form of the protease with the commercially available assays. In many cases the findings are based on up- or down-regulated expression of MMP genes, which do not necessarily translate into an increased presence of active proteases. This is the main limitation in the use of MMPs and TIMPs as markers of renal fibrosis. Given the functional complexity of the MMPs, it is likely that they themselves might not be suitable biomarkers of renal fibrosis.

### Protein fingerprint technology

**Figure 2:**
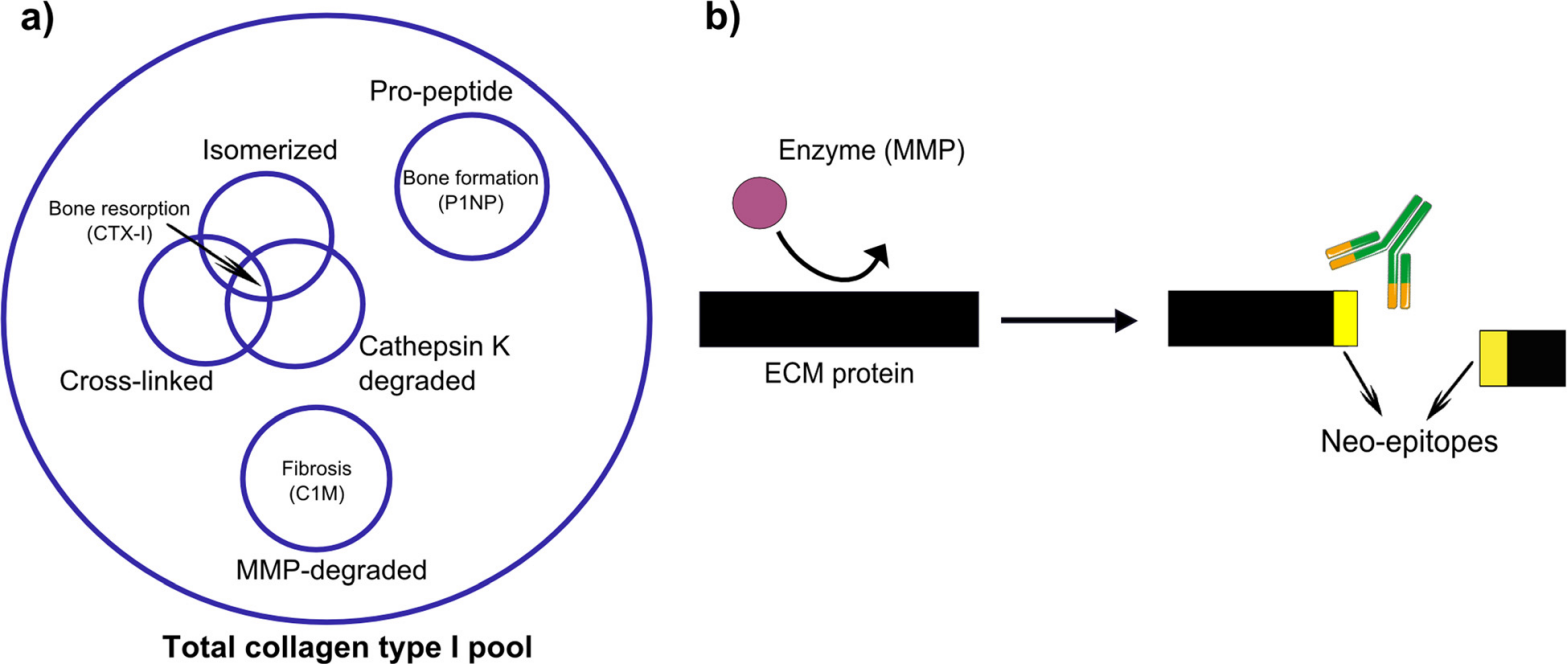
**Neo-epitope markers for ECM remodelling. a)** Neo-epitopes of collagen type I generated by different post-translational modifications provide more information than the measurement of total collagen type I. **b)** Formation of detectable neo-epitopes generated by cleavage of ECM proteins by specific proteases. ECM, extracellular matrix.

A highly regulated equilibrium between synthesis and degradation of ECM proteins is required to maintain tissue homeostasis. A disruption of this equilibrium is at the base of pathological processes such as fibrosis [104]. The measurement of the ECM remodelling rate, represented by end-products of ECM proteins in the biological fluids [116], can give an indication on the disease activity and progression. The peptides generated by specific protein degradation by MMPs or other proteases involved in a specific disease provide a unique fingerprint for a particular disease [117]. This approach is called protein fingerprint (Figure 2a). Compared to measurement of the intact/whole protein, the measurement of such modified ‘fingerprint’ peptides are likely to be more sensitive markers of pathology. This is because only the action of a specific protease (or other post-translational modifications) on a specific protein that is accumulated in a particular diseased tissue can generate the new N- or C-terminal, namely the neo-epitope.

The peptides originating from the protease-mediated degradation of the ECM may be small enough to be released in circulation or urine. There they can be detected by antibodies raised specifically to react against the neo-epitope (Figure 2b). Other post-translational modifications, for example isomerization, citrullination, glycosylation and cross-linking can also originate from neo-epitopes to be used for protein fingerprint [118], but will be not discussed here. Markers reflecting ECM remodelling can not only identify and quantify a pathological process within the organ of interest, but can potentially describe the disease activity. This might for example help to segregate the patients that progress faster with the disease. Markers reflecting the disrupted ECM turnover might detect tissue modifications, which happen in the first stages of the disease when the pathological process can possibly still be reversed.

**Figure 3:**
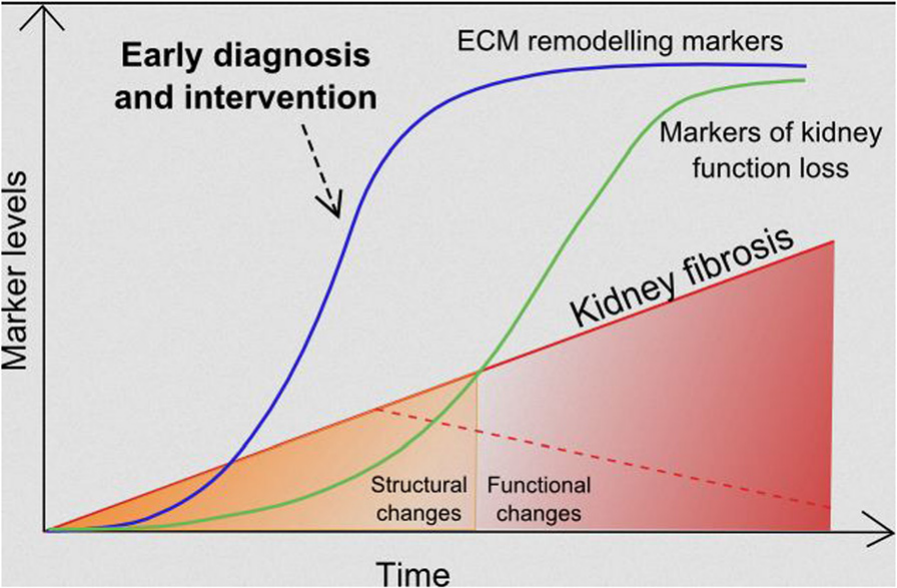
**Biomarkers of ECM remodelling may identify molecular processes occurring in the early phases of fibrogenesis, giving the opportunity for early intervention in stages in which the disease is still reversible.** The development of fibrosis is schematically indicated as linear for simplicity. ECM, extracellular matrix.

As outlined above, surprisingly very little data exists on the use of ECM, the principal underlying structure of fibrotic tissue, as a source of biomarkers of renal fibrosis. Such biomarkers could identify the early modifications that lead to renal fibrosis and could allow early treatment, helping in the resolution of fibrosis. Figure 3 illustrates the possible advantages of markers of structural changes over markers of loss of kidney function.

Neo-epitopes of different types of collagen (type I, II, III, IV, V and VI collagen), proteoglycans (biglycan and versican) and elastin have already proven to be biomarkers of connective tissue diseases, such as osteoarthritis [119] or organ fibrosis, in both animal models and clinical studies [120-130].

Experimental evidence in well-characterized animal models showed that neo-epitope fragments of collagen type I (C1M), III (C3M), IV (C4M) and V (C5M), biglycan (BGM) MMP-mediated degradation, and of collagen type III (Pro-C3), IV (P4NP 7S) and V (P5NP) formation were markers of liver fibrosis [120,126-129,131]. These markers (except BGM) also showed a promising potential for monitoring the efficacy of the treatment with statins in an experimental model of liver fibrosis [132]. C3M was elevated in urine of mice treated with bleomycin to induce skin fibrosis compared to the controls, showing a potential use of this marker in skin fibrosis [125].

Clinical studies showed that the markers BGM, elastin MMP-generated neo-epitope fragment (ELM) C1M, C3M, C4M C5M, collagen type VI MMP-generated neo-epitope fragment (C6M), Pro-C3 and P4NP 7S were associated with portal hypertension in patients with cirrhosis, reflecting the degree of liver dysfunction [123]. A marker of MMP-mediated versican degradation (VCANM) was elevated in plasma of patients suffering from different cardiovascular diseases [130]. Promising clinical results were also obtained in lung fibrosis: the previously mentioned ELM [124] C1M, C3M, C4M, C5M and C6M [121] could separate patients affected by chronic obstructive pulmonary disease (COPD) and idiopathic pulmonary fibrosis (IPF) from healthy individuals in a small observational cohort.

As the mechanisms of kidney, liver and lung fibrosis share common features and involve similar ECM proteins, the successful biomarkers identified in these pre-clinical and clinical studies are also likely to prove valuable in renal fibrosis, as a first study in kidney patients suggests. Plasma levels of P4NP 7S were significantly associated with mortality in ESRD patients undergoing hemodialysis [122]. Specifically, the patients in the highest quartile of P4NP 7S plasma levels had an increased risk of death compared to the patients in the other quartiles. The high plasma levels of this marker were considered a sign of accelerated systemic fibrosis in ESRD patients with the worst prognosis. These results confirm the high value of collagen type IV as a prognostic marker in kidney diseases demonstrated by the previously described studies. The before mentioned results were obtained in urine and using an assay based on polyclonal antibodies, while in this study, an assay using a specific monoclonal antibody for the α1 chain of the P4NP 7S domain of collagen type IV was used to detect collagen type IV in plasma [133].

The main limitation of this technique in kidneys is that neo-epitope peptides coming from organs other than kidneys can also contribute to the pool of neo-epitopes detected in serum or plasma. Urine is a more suitable matrix to find protein fragments originating in the kidney. However, the detection of protein fragments in urine can be biased by the altered GFR during the late stages of CKD: lower or higher levels of the markers cannot be a result of lower or higher remodelling, but of impaired excretion. Furthermore, the urinary concentration of the markers can be altered by non-selective proteinuria in proteinuric kidney disease. The picture is further complicated by the frequent presence of co-morbidities affecting other organs in the presence of kidney diseases, or even causing kidney diseases in the first place.

The challenge to identify a disease- and/or organ-specific and sensitive biomarker for renal fibrosis might be met by narrowing the selection of neo-epitopes to ECM protein or protein isoforms that are most exclusively expressed in kidneys and the action of a protease whose expression is up-regulated specifically during the pathogenesis of renal fibrosis.

## Conclusions

The identification of reliable biomarkers for early diagnosis and prognosis of renal fibrosis is of paramount importance. The perfect biomarker for kidney fibrosis should be non-invasive, specific, involved in the mechanisms of fibrosis, with low (or no) background in healthy individuals and able to reflect treatment effects. Several molecules implicated in the mechanisms of fibrosis have been proposed as biomarkers, but none of them have been validated and accepted in clinical practice yet. In this review we have proposed a new perspective, introducing the possible use of ECM protein fingerprint as a source of novel biomarkers for renal fibrosis.

## Abbreviations

ACR: Albumin to creatinine ratio; ADAM: A disintegrin and metalloproteinase; ADAMTS: A disintegrin and metalloproteinase with thrombospondin motifs; ADMA: Asymmetric dimethylarginine; ADPKD: Autosomal dominant polycystic kidney disease; ANCA: Anti-neutrophil cytoplasmic antibody; BGM: MMP-generated neo-epitope fragment of biglycan; BUN: Blood urea nitrogen; C1M: MMP-generated neo-epitope fragment of collagen type I; C3M: MMP-generated neo-epitope fragment of collagen type III; C4M: MMP-generated neo-epitope fragment of collagen type IV; C5M: MMP-generated neo-epitope fragment of collagen type V; C6M: MMP-generated neo-epitope fragment of collagen type VI; CKD: Chronic kidney disease; COPD: Chronic obstructive pulmonary disease; CRP: C-reactive protein; DN: Diabetic nephropathy; ECM: Extracellular matrix; eGFR: Estimated glomerular filtration rate; ELM: MMP-generated neo-epitope fragment of elastin; ESRD: End-stage renal disease; FGF: Fibroblast growth factor; FGF-R1: Fibroblast growth factor receptor 1; GAG: Glycosaminoglycan; GBM: Glomerular basement membrane; GFR: Glomerular filtration rate; GGT: Gamma-glutamyl transpeptidase; IgAN: IgA nephropathy; IGF: Insulin-like growth factor; IPF: Idiopathic pulmonary fibrosis; KIM-1: Kidney injury molecule 1; L-FABP: Liver-type fatty acid-binding protein; MESA: Multi-Ethnic Study of Atherosclerosis; MeSH: Medical Subject Headings; MMP: Matrix metalloproteinase; MRE: Magnetic resonance elastography; NAG: N-acetyl-beta-D-glucosaminidase; NGAL: Neutrophil gelatinase-associated lipocalin; NIH: National Institutes of Health; P4NP 7S: Collagen type IV fragment belonging to the 7S domain; P5NP: Collagen type V pro-peptide; PAI-1: Plasminogen activator inhibitor-1; PDGF: Platelet-derived growth factor; PIIINP: Collagen type III N-terminal pro-peptide; Pro-C3: Propeptide of collagen type III; PSTC: Predictive Safety Testing Consortium; SLRP: Small leucine-rich proteoglycan; T4C: Collagen type IV to creatinine ratio; TGF-β1: Transforming growth factor beta 1; TIMP: Tissue inhibitors of metalloproteinase; TNFR: Tumor necrosis factor receptor; uPIIINP/Cr: Urinary PIIINP to creatinine ratio; UUO: Unilateral ureteral obstruction; VCANM: MMP-mediated versican degradation fragment.

## Competing interests

FG, MK and DL are full-time employees at Nordic Bioscience, Herlev, Denmark. Other authors have no competing interests.

## Authors’ contributions

FG, AM, MK and DL conceived and designed the review. FG, AM and PB carried out the literature research and drafted the manuscript. PB, DL and MK critically revised the manuscript for important intellectual content. All authors read and approved the final manuscript.

## Supplementary Material

Additional file 1: Table S1 Pre-clinical and clinical experimental evidence of involvement of extracellular matrix (ECM) protein and proteases in kidney disease [14,53,55-59,63,69-73,77-79,83-86,90], [94,97,99-101,112,113,115,134-154]. Click here for file
