# Supplementary material for: The extracellular matrix in the kidney: a source of novel non-invasive biomarkers of kidney fibrosis?
Source: Fibrogenesis Tissue Repair. 2014 Mar 28;7:4. doi: 10.1186/1755-1536-7-4 (PMC3986639; doi:10.1186/1755-1536-7-4)
Supplement: Additional file 1: Table S1 — Pre-clinical and clinical experimental evidence of involvement of extracellular matrix (ECM) protein and proteases in kidney disease [14,53,55-59,63,69-73,77-79,83-86,90], [94,97,99-101,112,113,115,134-154]. [file 1755-1536-7-4-S1.doc]

**Additional file 1: Table S1: Pre-clinical and clinical experimental evidences of involvement of ECM protein and proteases in kidney disease.**

| **Protein** | **Experimental model** | **Detection method** | **Result** | **Ref** |
| --- | --- | --- | --- | --- |
| **MMP-1** | Rat UUO | Collagenase activity assays | Slight increase of activity until day 3 and decrease afterwards | [112] |
|  | SNx in rat | NB | Increased mRNA at day 7 | [73] |
|  |  | Zymography | Increased activity at day 90 |  |
| **MMP-2** | Rat UUO | Gelatinase activity assay | Increased activity until day 6 and decrease afterwards | [112] |
|  |  | Zymography, WB | Increased expression and activity | [134,135] |
|  |  | RT-PCR | Upregulated mRNA, peak at day 3 | [134] |
|  | I-R injury | RT-PCR and ELISA | Upregulated after 8 weeks, and down-regulated at 16 weeks when TIMP-1 expression increases | [136] |
|  | Spontaneous hypertensive rats | IHC and zymography | Increased levels in cortex | [137,138] |
|  | Transgenic mice | RT-PCR | Upregulated mRNA | [139] |
|  | Rat model for CAN | Zymography | Increase of proMMP-2 and active MMP-2 | [139] |
|  | STZ-induced T1D rat | NB and in-situ hybridation | Downregulated mRNA and expression | [140] |
|  | Goto-Kakizaki T2D rat | Gelatin zymography, fluorogenic gelatinase assay, immunoblotting | Increased activity and protein levels | [141] |
|  | SNx in rat | NB | Increased mRNA at day 7 | [73] |
|  |  | Zymography | Increased activity at day 90 |  |
|  | Polycystic kidney disease in rat | Zymography | Decreased activity | [113] |
|  |  | WB | Decreased protein levels |  |
|  |  | NB | Downregulated mRNA |  |
|  | Human DN | RT-PCR | Downregulated mRNA (glomeruli) | [69] |
| **MMP-3** | Human DN | NB | Downregulated mRNA (glomeruli) | [69] |
|  | STZ-induced diabetes in rats | High-resolution in situ hybridization | Decreased activity (glomeruli) |  |
| **MMP-7** | Human ADPKD | NB | Present in apical/luminal cells of atrophic tubules and epithelial cells | [115] |
|  | Mice folic acid-induced fibrosis | NB | mRNA present 48h post-induction |  |
|  | Mice UUO | IHC | Protein present in apical/luminal cells of tubules, epithelial cells of medulla and cortex |  |
|  | Spontaneous hypertensive rats | Zymography | Increased levels in medulla at week 6 | [137] |
| **MMP-9** | Rat UUO | Gelatinase activity assay, NB | Increase of activity until day 6 and decrease afterwards  Initial peak of mRNA at day 7, then decrease to control levels | [112] |
|  | Spontaneous hypertensive rats | Zymography | Increased levels in cortex and medulla at week 6 | [137] |
|  | Rat model for CAN | Zymography | Increased proMMP-9 and active MMP-9 | [139] |
|  | Goto-Kakizaki T2D rat | Immunoblotting | Increased protein levels | [141] |
|  | SNx in rat | NB | Increased expression | [73] |
|  | STZ-induced diabetes in rats | RT-PCR and zymography | Decreased activity and mRNA levels | [69] |
| **ADAM17** | IF/TA in humans | RT-PCR and *in situ* hybridation on renal biopsies | Upregulated mRNA in tubules, capillaries and glomeruli. *De novo* expression in mesangium | [99] |
| **ADAM19** | Various human kidney diseases | *In situ* hybridation on renal biopsies | Upregulated mRNA in distal tubules and endothelial cell. *De novo* expression in proximal tubules and glomerular mesangium | [100] |
|  | Human chronic allograft nephropathy | RT-PCR and *in situ* hybridation on nephrectomies | Upregulated mRNA on SMCs, distal tubuli and glomerulosclerotic regions | [101] |
| **TIMP-1** | Rat UUO | WB | Increased expression from day 9 | [112] |
|  | I-R injury | RT-PCR and ELISA | Increased expression | [136,142] |
|  | SNx in rat | NB | Upregulated mRNA at day 7 | [73] |
|  |  | WB | 4-fold increased expression at day 90 |  |
|  | Human interstitial fibrosis and rat models | NB | mRNA and protein levels elevated in interstitium | [14] |
|  | Rats with protein-overload proteinuria | In-situ hybridation | TIMP-1 transcription by tubular and interstitial cells | [143] |
|  | Polycystic kidney disease in rat | NB | Upregulated mRNA (tubules) | [113] |
|  | Uninephrectomized rats, BSA injected | NB | Upregulated mRNA (260% increase) | [14] |
|  | Murine BSA-induced overload proteinuria | WB | Increased expression | [14] |
|  | Diet-induced hypercholesterolemia in rats | NB | Upregulated mRNA after 12weeks | [144] |
|  | Rat UUO | NB | Upregulated mRNA 12h after ligation | [145] |
| **TIMP-2** | SNx in rat | NB | Upregulated mRNA at day 120 | [73] |
|  | Polycystic kidney disease in rat | NB | Upregulated mRNA (tubules) |  |
| **TIMP-3** | Rat model for CAN | Zymography | Decreased expression | [139] |
|  | SNx in rat | NB | Downregulated mRNA at day 30 | [73] |
|  |  | WB | 4-fold increased expression at day 90 |  |
| **TIMP-4** | Spontaneous hypertensive rats | Zymography | Increased levels in medulla at week 6 | [137] |
| **Col I** | SNx in rat | NB | Upregulated mRNA at day 7 | [73] |
|  | ADPKD patients | Urine proteome | Elevated α1 chain levels in urine | [78] |
|  | Human glomerular diseases | IHC | Protein deposition in the urinary space of fibrotic glomeruli, tubulointerstitium and arterial walls. | [79] |
|  |  | In situ hybridation | mRNA synthesis detectable in glomeruli and peritubular cells resembling myo/fibroblasts. |  |
|  | Rabbit UUO | IHC, mRNA quantification and NB | Upregulated mRNA and increased deposition between days 3-7 | [77] |
|  | Rat UUO | IHC, mRNA extraction and spectrophotometry, Sircol assay, WB | Increased mRNA and protein levels | [146] |
|  | Mice UUO | Sirius Red staining, IHC | Increased protein levels 5 days after UUO | [147] |
|  | Human DN | EM, immunofluorescence microscopy on biopsies | Deposition of newly synthesized Col I in mesangial area | [148] |
|  | Rat diet-induced hypercholesterolemia | Laser densitometry | Upregulated pro-Col I mRNA (30% increase) at week 12 | [144] |
| **Col III** | SNx in rat | NB | Upregulated mRNA at day 7 | [73] |
|  | Various human kidney diseases | IHC on kidney biopsies | Significantly higher expression in diseased compared to control tissue | [84] |
|  | Rabbit UUO | IHC, mRNA quantification and NB | Upregulated mRNA between days 3-7 | [77] |
|  | Rat UUO | IHC | Increased protein levels | [146] |
|  | Human DN | IHC | Expression in the extraglomerular interstitium in diseased but not in healthy samples | [69] |
|  | Rat diet-induced hypercholesterolemia | Laser densitometry | Upregulated pro-Col III mRNA (70% increase) at week 12 | [144] |
|  | CKD patients | Urine PIIINP/Creatinine ratio | Correlation with interstitial fibrosis and eGFR | [83] |
| **Col IV** | SNx in rat | NB | Upregulated mRNA at day 7 | [73] |
|  | Human IgAN | Sandwich EIA | Elevated excretion in urine | [72] |
|  | Diabetic *db/db* mice | NB, EIA | Increased mRNA and protein levels | [71] |
|  | Rat UUO | IHC | Increased protein levels | [146] |
|  | Mice UUO | Sirius Red staining, RT-PCR | Increased mRNA and protein levels | [147] |
|  | Mice Alport’s syndrome model | Microarray, IHC | Knock out of Col4A3 gene causes progressive interstitial fibrosis | [149] |
|  | Various human kidney diseases | IHC on kidney biopsies | Positive staining in diseased but not in control patients | [84] |
|  | Human DN | WB, ELISA, IHC, Proteomics | Upregulated α1(IV) chain in GMC  Upregulated α3(IV) and α5(IV) chains in GEC  Elevated excretion in urine (type 1 diabetes)  Elevated excretion in urine (type 2 diabetes)  Decreased presence of fragments of α1 (IV) chain in urinary peptidome | [55-57,59,150] |
|  | Murine models for diabetes | IHC, *in situ* hybridation | Increased deposition of α1 and α2 chains in the mesangial area  Increased glomerular expression and accumulation | [69] |
|  | Human chronic transplant nephropathy | IHC | Increased deposition in the mesangial matrix and GBM | [53] |
|  | Membranous nephropathy and ANCA-associated glomerulonephritis | ELISA | Elevated excretion in urine | [58] |
| **Col V** | Various human kidney diseases | IHC on kidney biopsies | Increased accumulation in diseased compared to control tissue and correlation with disease severity | [84] |
|  | Human DN | IHC, Proteomics | Increased presence in mesangium  Decreased presence of fragments of Alpha 1 (V) chain in urinary peptidome | [59,69] |
| **Col VI** | Various human kidney diseases | IHC on kidney biopsies | Increased accumulation in diseased compared to control tissue | [84] |
| **Decorin** | Glomerulonephritis induced by ATS in rats | IP | Increased expression in glomeruli 4days after ATS | [85] |
|  | Rat UUO | WB | Upregulated mRNA after 24h  Elevated protein expression after 96h in renal cortex | [90] |
|  | Human glomerular diseases | IHC | Deposition around glomeruli and tubules at 96h, and in the interstitium at 168h  Deposition in amyloid deposits of glomeruli, tubulointerstitium and arterial walls.  Deposition in the urinary space of fibrotic glomeruli. | [79] |
|  | Various human kidney diseases | In situ hybridation | mRNA detectable in glomeruli and peritubular cells resembling myo/fibroblasts. Protein localized in the adventia of arterial vessels | [84] |
|  |  | IHC on kidney biopsies | Increased deposition in diseased compared to control tissue |  |
|  | Human membranous nephropathy | IHC in kidney biopsies | Accumulation in fibrotic tubulointerstitium. Weak staining in glomeruli | [70] |
|  | Human IgAN | RT-PCR and immunofluorescence on kidney biopsies | Upregulated mRNA (45.1% increase in glomeruli).  Protein expression in sclerotic glomeruli, but no difference in tubules compared to controls | [63] |
| **Biglycan** | Glomerulonephritis induced by ATS in rats | IP | Increased expression in glomeruli after 4 days | [85] |
|  | Human glomerular diseases | IHC | Protein deposition in the urinary space of fibrotic glomeruli.  Protein localized in the adventia of arterial vessels | [79] |
|  | Human membranous nephropathy | In situ hybridation | mRNA detectable in glomeruli and peritubular cells resembling myo/fibroblasts. | [70] |
|  |  | IHC in kidney biopsies | Accumulation in fibrotic tubulointerstitium. Weak staining in glomeruli |  |
|  | Mice UUO | NB | Upregulated mRNA after 7 days, previous to macrophage infiltration | [151] |
|  | Human IgAN | RT-PCR and immunofluorescence on kidney biopsies | Upregulated mRNA (136.6% increase in glomeruli) | [63] |
| **Perlecan** | Human IgAN | RT-PCR and immunofluorescence on kidney biopsies | Upregulated mRNA (87% % increase in glomeruli)  Downregulated mRNA (38.2% decrease in tubules)  Increased protein expression in IgAN glomeruli | [63] |
| **Syndecan-1** | Human IgAN | RT-PCR and immunofluorescence on kidney biopsies | 71.1% decrease in gene expression in glomeruli. No difference in tubular expression compared to controls | [63] |
| **Fibronectin** | SNx in rat | NB | Upregulated mRNA at day 60 | [73] |
|  | Diabetic *db/db* mice | NB, EIA | Increased mRNA and protein levels | [71] |
|  | Rat UUO | IHC | Increased protein levels | [146] |
|  | Mice UUO | Sirius Red staining, RT-PCR, IHC | Increased mRNA and protein levels | [147] |
|  | ADPKD patients | Urine proteome | Elevated levels of α1 chain in urine | [78] |
|  | Glomerulonephritis induced by ATS in rats | IP | Increased expression in glomeruli from 4 days after | [85] |
|  | Goto-Kakizaki T2D rat | Immunoblotting | Increased protein levels | [141] |
|  | Human DN | Histology, IHC | Increased glomerular expression and accumulation | [69] |
| **Fibromodulin** | Human membranous nephropathy | IHC in kidney biopsies | Accumulation in fibrotic tubulointerstitium. Weak staining in glomeruli | [70] |
| **Lumican** | Human membranous nephropathy | IHC in kidney biopsies | Accumulation in fibrotic tubulointerstitium. Weak staining in glomeruli | [70] |
| **Laminin** | SNx in rat | NB | Upregulated mRNA at day 60 | [72] |
|  | Various human kidney diseases | IHC on kidney biopsies | Positive staining in diseased but not in control patients | [84] |
| **HSPGs** | SNx in rat | NB | Upregulated mRNA at day 60 | [73] |
|  | Various human kidney diseases | IHC on kidney biopsies | Positive staining in diseased but not in control patients | [84] |
|  | Human DN | Immunofluorescence staining, IEM | Decreased presence in GBM | [69] |
|  | Murine models of diabetes | IHC | Decreased expression | [69] |
| **Versican** | Human proteinuric nephropathies | IHC on renal biopsies | Expression increases in areas of marked interstitial fibrosis | [97] |
|  | Mice proliferative glomerulonephritis model | RT-PCR | Upregulated mRNA after 14d |  |
|  | Rat ADR nephritis | RT-PCR, IHC | Upregulated mRNA and protein after 21d |  |
|  | Rat PHN | RT-PCR, IHC | Upregulated mRNA and protein compared to controls |  |
|  | Human DN | RT-PCR on kidney biopsies | Upregulated mRNA expression (1,4-fold elevated) compared to healthy controls | [152] |
| **Thrombospondin1** | Rat models of glomerulonephritis (Anti-Thy1, PAN and PHN) | IHC and *in situ* hybridation | Upregulated mRNA and protein in the interstitium 5-12 days before fibrosis appeared | [86] |
| **Hyaluronan** | STZ-induced diabetic rats | RT-PCR | Upregulated mRNA in renal papilla (3-fold higher than in healthy control) | [153] |
|  | Rejected human kidneys | Biotin-labeled hyaluronan binding protein | Accumulation primarily in the cortex and sclerotic vessels (it is in expressed the medulla in healthy kidneys) | [154] |
|  | Different human kidney diseases (DN, I-R injury, transplant-rejected kidneys, and acute tubular necrosis) | IHC and *in situ* hybridation | Increased expression in the interstitium | [94] |
|  | ADPKD patients | iTRAQ, MS and MRM | Elevated urine levels | [78] |

**List of abbreviations:**

I-R: ischemia-reperfusion

CAN: chronic allograft nephropathy

STZ: streptozotocin induced type 1 diabetes

T1/2D: Type 1/2 diabetes

SNx: subtotally nephrectomised

N/WB: Northern/Western Blot

HSPGs: heparan sulphate proteoglycans

MCP-1: monocyte chemoattractant protein 1

ADPKD: autosomic dominant polycystic kidney disease

Anti-Thy1: rats with mesangial proliferative nephritis induced by injection of anti-rat thymocyte plasma

PHN: Experimental membranous nephropathy

PAN: Aminonucleoside nephrosis

ATS: anti thymocyte serum

DN: Diabetic nephropathy

AIN: acute interstitial nephritis

*db/db* mice: diabetes type 2 model

PIIINP: N-terminal peptide of collagen type III

rGFR: estimated glomerular filtration rate

EM: electron microscopy

IEM: immunoelectron microscopy

EIA: enzyme immunoassay

GEC: glomerular endothelial cells

GMC: glomerular mesangial cells

RT-PCR: reverse transcription polymerase chain reaction

iTRAQ: Isobaric tag for relative and absolute quantitation

MS: mass spectrometry

MRM: multiple reaction monitoring

TF/TA: interstitial fibrosis and tubular atrophy

SMCs: smooth muscle cells
